# Could AGE/RAGE-Related Oxidative Homeostasis Dysregulation Enhance Susceptibility to Pathogenesis of Cardio-Metabolic Complications in Childhood Obesity?

**DOI:** 10.3389/fendo.2019.00426

**Published:** 2019-06-28

**Authors:** Domenico Corica, Tommaso Aversa, Rosaria Maddalena Ruggeri, Mariateresa Cristani, Angela Alibrandi, Giorgia Pepe, Filippo De Luca, Malgorzata Wasniewska

**Affiliations:** ^1^Department of Human Pathology of Adulthood and Childhood, Unit of Pediatrics, University of Messina, Messina, Italy; ^2^Department of Clinical and Experimental Medicine, Unit of Endocrinology, University of Messina, Messina, Italy; ^3^Department of Chemical, Biological, Pharmaceutical, and Environmental Sciences, University of Messina, Messina, Italy; ^4^Department of Economics, University of Messina, Messina, Italy

**Keywords:** oxidative stress, advanced glycation end-products, cardiometabolic risk, sRAGE, AGEs/sRAGE-ratio, advanced oxidation-protein products

## Abstract

**Background:** Advanced glycation end-products (AGEs) and their cell receptor (RAGE) are involved in the pathophysiology of cardio-metabolic diseases. Interaction of AGEs with RAGE results in increased generation of oxygen radicals and pro-inflammatory cytokines. Circulating soluble RAGE (sRAGE) interacts with AGEs in order to counterbalance the negative effects of AGEs-RAGE interaction.

**Objectives:** To define factors influencing AGEs, sRAGE, AGEs/sRAGE-ratio, and advanced oxidation-protein products (AOPPs) levels and to investigate changes in oxidative balance among overweight/obese children.

**Materials and methods:** Cross-sectional, one Center, case-control study included 41 overweight and obese children aged between 5 and 16 years and 36 lean matched controls. Inclusion criteria were: BMI ≥ 1 SD; term birth; no genetic or endocrine causes of obesity; no associated chronic diseases neither chronic therapies. All patients underwent clinical and biochemical investigations (lipid and glucose profiles, liver, renal and thyroid function tests, uric acid, C-reactive protein (CRP), AGEs, sRAGE, and AOPPs serum concentrations). Significance was established at 0.050.

**Results:** AOPPs, AGEs/sRAGE-ratio, HOMA-IR, triglycerides, triglycerides/HDL-ratio, total cholesterol (TC)/HDL-ratio, atherogenic-index of plasma (AIP), uric acid, CRP were significantly higher, whereas sRAGE and HDL were significantly lower in overweight/obese children than controls. sRAGE was significantly negatively correlated with BMI SD, TC/HDL-ratio, CRP, AOPPs, and positively with HDL. AGE/sRAGE-ratio and AOPPs were significantly positively correlated with BMI SD, TC/HDL-ratio, AIP, CRP, and negatively with HDL. BMI SD was independently associated with AGEs/sRAGE-ratio (*B* = 0.06; *p* = 0.008), AOPPs (*B* = 0.13; *p* = 0.02), and sRAGE (*B* = −73.18; *p* = 0.000).

**Conclusions:** We demonstrated, for the first time in a pediatric cohort, a significant higher value of AGEs/sRAGE-ratio among overweight/obese children, expression of a relative shift to oxidant from anti-oxidant factors, suggesting an AGE/RAGE-related oxidative homeostasis dysregulation that could enhance susceptibility to oxidative/inflammatory tissues damage. Severity of overweight, influencing the increase of oxidative stress in human organism and even in children, may contribute to the pathogenesis of long-term cardiovascular and metabolic alterations.

## Introduction

Childhood obesity and its correlated comorbidities, such as insulin-resistance (IR), fatty liver, type 2 diabetes, metabolic syndrome, are among the most important health issues worldwide (1, 2). The pathogenesis of adiposity-related cardio-vascular and metabolic precocious alterations is the result of concurrent pathways of inflammation, apoptosis, and oxidative stress (3, 4). The oxidative stress pathways related to advanced glycation end-products (AGEs) generation were well-studied in adults and they were implicated in inflammation, endothelial dysfunction, IR, glucose homeostasis alterations and metabolic syndrome (5–7). AGEs result from non-enzymatic glycation and oxidation of proteins, lipids, and nucleic acids. AGEs and their transmembrane cell receptor (RAGE) have been involved in the pathophysiology of cardiovascular and metabolic diseases (7). Interaction of AGEs with RAGE results in both increased generation of oxygen radicals and increased expressions of pro-inflammatory cytokines (7). Circulating soluble RAGE (sRAGE) is able to interact with AGEs, in order to counterbalance the negative effects of AGEs-RAGE interaction (7). AGEs/sRAGE-ratio has been suggested to be a reliable marker of oxidative state (8) as well as advanced oxidation protein products (AOPPs) (9). In children, the relationships between oxidative stress markers and clinical and biochemical variables considered markers of cardio-metabolic risk are not widely investigated and the results so far available are contrasting.

Purposes of this study are to define factors influencing AGEs, sRAGE, AGEs/sRAGE-ratio, and AOPPs levels and to investigate changes in oxidative balance in a cohort of overweight and obese children and adolescents compared to controls.

## Materials and Methods

### Subjects

Study population included 41 Caucasian overweight and obese children aged between 5 and 16 years (Group A) and 36 Caucasian, healthy, lean (BMI SD < 1), age and sex-matched controls (Group B). Patients, who referred to the Outpatient Clinic of Pediatric Endocrinology at the University of Messina (Italy), were selected according to the following criteria: BMI ≥ 1 SD according to the WHO growth references (10), full-term birth appropriate for gestational age; no genetic or endocrine pathological causes of obesity; no associated chronic diseases; no chronic pharmacological therapies.

This study was approved by Ethics Committee of Messina. Written informed consent was obtained from parents or legal tutors.

### Methods

At entry, family history and personal anamnesis were obtained and all subjects underwent physical examination, including staging of puberty and anthropometric assessment (height, weight, waist circumference, BMI) according to standard procedures, as previously described (11, 12). Waist-to-height-ratio (WHtR) was measured in patients of group A. Furthermore, the following diagnostic investigations were performed: (1) serum lipid profile (total cholesterol, HDL, LDL, triglycerides), uric acid, C-reactive protein (CRP), thyroid, kidney and liver function tests were measured, in the fasting state, with routine methods using commercial kits, as previously described (11); (2) oral glucose tolerance test (OGTT) was performed, in patients of group A only, with standard method (1.75 g/kg of body weight, up to a maximum of 75 g), measuring glucose and insulin serum levels at baseline and during OGTT (at 0, 30′, 60′, 90′, and 120′ min); (3) homeostasis model assessment of insulin resistance (HOMA-IR) (13), atherogenic index of plasma (AIP) (14), total cholesterol-to-HDL cholesterol ratio (TC/HDL-ratio) (15), and triglycerides-to-HDL cholesterol ratio (TG/HDL-ratio) (16) were assessed; (4) serum levels of oxidative stress markers (AGEs, sRAGE, and AOPPs) were measured.

We considered abnormal levels of triglycerides (TG), total cholesterol (TC), LDL, HDL according to the National Cholesterol Education Panel (17): TC > 170 mg/dl, LDL > 130 mg/dl, HDL < 40 mg/dl, TG > 110 mg/dl. Fasting glucose >100 mg/dl and fasting insulin >15 μUI/ml were assessed as abnormal. To define the other indices of cardio-metabolic risk as abnormal, the following cutoffs were considered: HOMA-IR >2.5 (13), AIP > 0.11 (14), TC/HDL-ratio ≥ 5.6 (15), TG/HDL-ratio >1.25 (16).

#### Oxidative Stress Markers Assessment

Serum levels of total sRAGE were measured by quantitative enzyme immunoassay technique, the RayBio® Human RAGE ELISA (RayBiotech, Norcross GA), according to the manufacturer's instructions. All assays were done in duplicate. The minimum detectable dose of Human RAGE was determined to be 3 pg/ml. The detection limit of the assay was 10 pg/ml. The intra or the inter-assay CV were <5 and <10%, respectively.

Serum levels of AGEs were measured by spectrofluorimetric detection, as previously described (18). Serum (25 μl, triplicates) was diluted 1:50 with phosphate-buffered saline (PBS) pH 7.4 and fluorescence intensity was recorded (λ emission = 370 nm, λ excitation = 440 nm; spectrofluorimeter Shimadzu, Japan). PBS solution was used as blank. Serum concentration of AGEs was normalized to the total protein amount determined by the Bradford assay and expressed in arbitrary units (AU) per gram of protein (AU/g prot).

Serum levels of AOPPs were estimated by spectrophotometric detection. Blood serum (100 μl) or same volume of chloramin T (0–100 μmol/l) for calibration were diluted 1:5 with PBS pH 7.4. Afterward, 25 μl of 1.16 M KI and 50 μl of acetic acid were added to the diluted solutions and absorbance was immediately measured at 340 nm (spectrophotometer Shimatzu, Japan). Concentration of AOPPs was expressed in chloramine T units (μmol eq Cl T/L).

### Statistical Analysis

Numerical data were expressed as median and range, categorical variables as absolute frequencies, and percentages. Most of the examined variables were not normally distributed, as verified by Kolmogorov–Smirnov test; consequently, the non-parametric approach was used. Comparison analysis was performed by Mann-Whitney test for numerical variables and by Chi-Square test for categorical variables (sex and pubertal stage). Spearman's correlation coefficient was evaluated for correlation analysis. Univariate and multivariate linear regression models were estimated to assess the possible dependence of sRAGE, AGEs, AGEs/sRAGE-ratio, and AOPPs (dependent variables) on some potential explicative variables such as BMI SD, WHtR, HOMA-IR, TC, TC/HDL-ratio, TG/HDL-ratio, AIP, LDL, HDL, TG, uric acid, CRP. In particular, multivariate model (by stepwise procedure) was estimated considering the following independent variables: BMI SD, HOMA-IR, TC/HDL-ratio, LDL, TG, uric acid, CRP.

Statistical analyses were performed using SPSS 17.0 for Window package. A *P*-value smaller than 0.050 was considered statistically significant.

## Results

The two groups were comparable for age, gender, and pubertal stage (all *P*-values >0.05). Median age of subjects belonging to group A was 10.5 (range 4–16 years); 51% of them were female and 51% were pre-pubertal. No significant gender or pubertal differences were detected in analyzed variables. In group A, 21% of subjects had BMI SD > 2.5, 43% between 2.5 and 2 SD, 36% between 2 and 1 SD. All subjects of group A presented abdominal obesity (WHtR ≥ 0.5; median 0.6/range 0.5–0.69). In group A, total cholesterol >170 mg/dl was seen in 11 patients (26.8%), and in two subjects (4.9%) it was >200 mg/dl. LDL was >130 mg/dl in two subjects (4.9%); HDL was <40 mg/dl in 10 children (24.4%). Triglycerides were >110 mg/dl in five children (12.2%). Two patients (4.9%) had fasting blood glucose >100 mg/dl. OGTT excluded diabetes in all patients and documented a condition of impaired glucose tolerance in three patients of group A (7.1%) who had normal fasting glucose. Hyperinsulinism was demonstrated in 17 patients (41.5%) at basal and OGTT evaluations. HOMA-IR was >2.5 in 17 subjects (41.5%), AIP was > 0.11 only in 4 (9.7%), TG/HDL-ratio was >1.25 in 27 patients (65.8%); no patients had TC/HDL-ratio ≥5.6.

**Figure 1:**
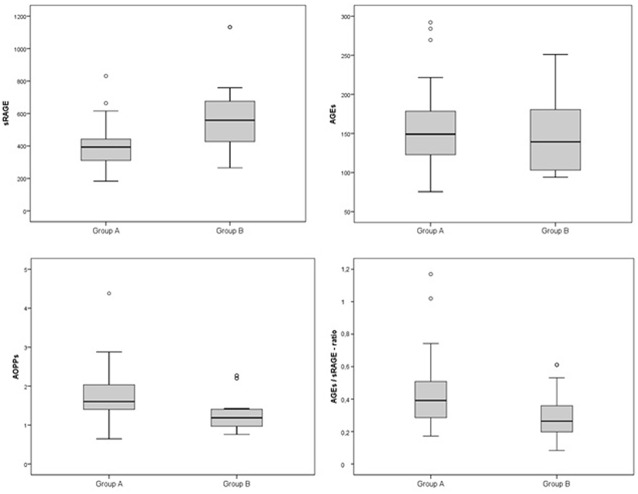
Box-plot of oxidative stress markers in groups A (overweight/obese) and B (controls).

**Figure 2:**
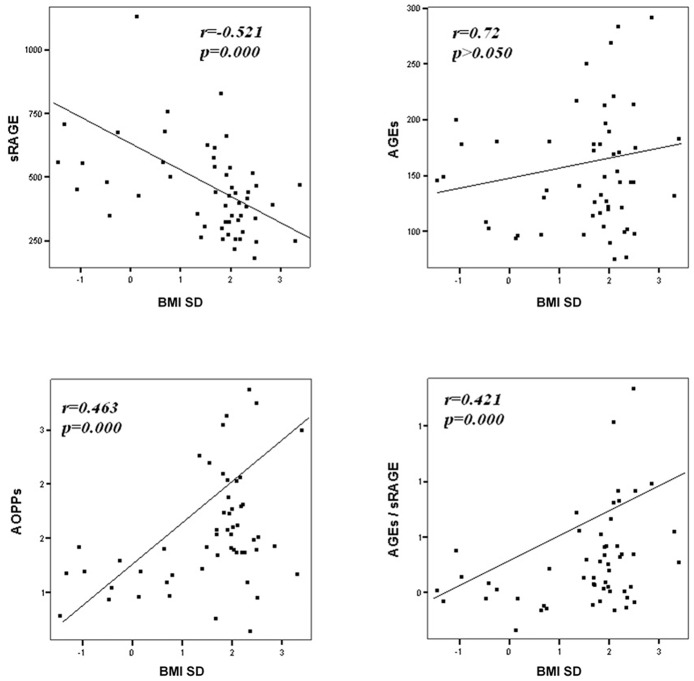
Correlation between oxidative stress markers and BMI SD.

Comparison analysis demonstrated significant differences of cardio-metabolic risk factors between group A and B (Table 1). Moreover, assessment of oxidative stress markers levels highlighted that sRAGE was significantly lower while AOPPs and AGEs/sRAGE-ratio were significantly higher in group A compared to group B (Table 1 and Figure 1). AGEs serum levels were higher in group A in comparison with group B, but the difference did not reach the statistical significance (Table 1 and Figure 1). Results of correlation analysis between oxidative stress markers and clinical and biochemical variables are reported in Table 2. In particular, BMI SD and TC/HDL-ratio were significantly negatively correlated with sRAGE and positively with AOPPs and AGEs/sRAGE-ratio (Figure 2). Moreover, AOPPs was significantly positively correlated with AGEs (*r* = 0.33, *p* = 0.003) and AGEs/sRAGE-ratio (*r* = 0.46, *p* = 0.000) and negatively with sRAGE (*r* = −0.33, *p* = 0.003); sRAGE and AGEs levels were not significantly related each other (*r* = 0.05, *p* = 0.66). The associations among sRAGE, AGEs, AGEs/sRAGE-ratio, AOPPs, and the main clinical and biochemical variables were analyzed by univariate linear regression analysis (Table 3). To investigate the independent effect of cardio-metabolic risk factors on oxidative stress markers levels a multivariate stepwise regression analysis was performed (Table 4). BMI SD was a strong predictor of AGEs/sRAGE-ratio, sRAGE, and AOPPs. Furthermore, CRP was associated with AOPPs and AGEs/sRAGE-ratio, and TG were associated with AOPPs and AGEs levels (Table 4).

**Table 1:** Comparison analysis of clinical data and cardio-metabolic risk factors between groups.

	**Group A (*n* = 41)**	**Group B (*n* = 36)**	***P*-value**
Age (years)	10.5 (5–16)	10.8 (5.5–15.4)	0.09
Male/Female (%)	48.8/51.2	33.3/66.7	0.17
Pre-pubertal/Pubertal (%)	51.2/48.8	48.1/51.9	0.08
BMI SD	2.1 (1.7–3.4)	0.41 (-1.4–1.7)	0.000
TC (mg/dl)	154 (123–238)	153 (112–193)	0.30
LDL (mg/dl)	88 (51–169)	78 (58–122)	0.07
HDL (mg/dl)	45 (25–119)	56 (35–69)	0.002
TC/HDL-ratio	3.42 (1.6–5.5)	2.59 (1.8–4.8)	0.000
TG (mg/dl)	71 (36–228)	60 (41–136)	0.01
TG/HDL-ratio	1.5 (0.6–6.5)	1.1 (0.7–3.6)	0.001
AIP	−0.2 (-0.6–0.5)	−0.3 (-0.5–0.2)	0.001
HOMA-IR	2.32 (0.7–6.3)	1.34 (0.8–3.5)	0.003
TSH (μlU/ml)	2.3 (0.6–7.98)	2.0 (1.3–5.3)	0.76
GPT (U/L)	17 (8–69)	14 (6–27)	0.000
GOT (U/L)	23 (16–37)	23 (17–32)	0.41
CRP (mg/dl)	0.2 (0.1–1.6)	0.1 (0.1–0.9)	0.008
Uric Acid (mg/dl)	4.6 (3.2–7.5)	4 (2.9–5)	0.000
sRAGE	393.3 (183.3–831.3)	558.3 (265.8–1132.3)	0.000
AGEs	149 (75.5–292)	139.3 (94.1–251.1)	0.36
AOPPs	1.6 (0.6–4.4)	1.2 (0.8–2.3)	0.000
AGEs/sRAGE-ratio	0.4 (0.2–1.2)	0.3 (0.1–0.6)	0.001

*Comparison analysis was performed by Mann-Whitney test for numerical variables (expressed as median and range) and by Chi-Square test for categorical variables (gender and pubertal stage)*.

*Group A, overweight and obese patients (BMI ≥ 1 SD); Group B, healthy matched controls (BMI <1 SD)*.

*TC, Total cholesterol; TG, Triglycerides; AIP, Atherogenic index of plasma; HOMA-IR, Homeostasis model assessment of insulin resistance; CRP, C-reactive protein; AGEs, Advanced glycation end-products; sRAGE, Circulating soluble AGE receptor; AOPPs, Advanced oxidation-protein products*.

**Table 2:** Correlation analysis among oxidative stress markers and cardio-metabolic risk factors (Spearman's test).

		**sRAGE**	**AGEs**	**AOPPs**	**AGEs/sRAGE**
BMI SD	*r*	−0.52	0.72	0.46	0.42
	*p*	**0.000**	0.53	**0.000**	**0.000**
WHtR	*r*	−0.2	0.07	−0.01	0.19
	*p*	0.21	0.65	0.95	0.22
TC	*r*	−0.10	0.08	0.09	0.19
	*p*	0.38	0.47	0.50	0.09
LDL	*r*	−0.12	0.23	0.16	0.28
	*p*	0.31	**0.047**	0.20	**0.015**
HDL	*r*	0.30	−0.17	−0.47	−0.33
	*p*	**0.008**	0.15	**0.000**	**0.004**
TC/HDL–ratio	*r*	−0.33	0.23	0.47	0.41
	*p*	**0.004**	**0.044**	**0.000**	**0.000**
TG	*r*	−0.05	0.15	0.52	0.14
	*p*	0.65	0.23	**0.000**	0.24
TG/HDL-ratio	*r*	−0.18	0.20	0.54	0.25
	*p*	0.12	n.s.	**0.000**	**0.026**
AIP	*r*	−0.18	0.19	0.54	0.25
	*p*	0.11	0.09	**0.000**	**0.028**
HOMA-IR	*r*	−0.20	−0.60	0.37	0.13
	*p*	0.10	0.63	**0.002**	0.28
CRP	*r*	−0.26	0.23	0.39	0.40
	*p*	**0.02**	**0.044**	**0.000**	**0.007**
Uric acid	*r*	−0.11	0.06	0.08	0.16
	*p*	0.35	0.58	0.50	0.17

*WHtR, Waist-to-height-ratio; TC, Total cholesterol; TG, Triglycerides; AIP, Atherogenic index of plasma; HOMA-IR, Homeostasis model assessment of insulin resistance; CRP, C-reactive protein; AGEs, Advanced glycation end-products; sRAGE, Circulating soluble AGE receptor; AOPPs, Advanced oxidation-protein products. Bold values indicate a statistically significant correlation*.

**Table 3:** Statistically significant associations at univariate linear regression analysis.

**Predictors**	**B**	***P*-value**
**AGEs**		
TC/HDL-ratio	14.07	0.02
LDL	0.59	0.03
Triglycerides	0.45	0.01
AIP	54.12	0.03
**sRAGE**		
BMI SD	−67.12	0.000
TC/HDL-ratio	−56.31	0.015
**AOPPs**		
BMI SD	0.202	0.000
HOMA-IR	0.123	0.03
Triglycerides	0.01	0.000
HDL	−0.012	0.02
TC/HDL-ratio	0.24	0.001
AIP	1.395	0.000
CRP	0.55	0.01
**AGEs/sRAGE-ratio**		
BMI SD	0.062	0.000
TC/HDL-ratio	0.065	0.008
CRP	0.214	0.002

*TC, Total cholesterol; AIP, Atherogenic index of plasma; HOMA-IR, Homeostasis model assessment of insulin resistance; CRP, C-reactive protein; AGEs, Advanced glycation end-products; sRAGE, Circulating soluble AGE receptor; AOPPs, Advanced oxidation-protein products*.

**Table 4:** Statistically significant associations at multivariate stepwise regression analysis for BMI SD, HOMA-IR, TC/HDL-ratio, LDL, Triglycerides, uric acid, CRP.

**Predictors**	**B**	***P*-value**
**AGEs**		
Triglycerides	0.618	0.01
**sRAGE**		
BMI SD	−73.18	0.000
**AOPPs**		
BMI SD	0.13	0.02
CRP	0.522	0.008
Triglycerides	0.010	0.000
**AGEs/sRAGE-ratio**		
BMI SD	0.06	0.008
CRP	0.160	0.03

*CRP, C-reactive protein; AGEs, Advanced glycation end-products; sRAGE, Circulating soluble AGE receptor; AOPPs, Advanced oxidation-protein products*.

## Discussion

To the best of our knowledge, this is the largest case-control pediatric study to simultaneously evaluate serum levels of sRAGE, AGEs, and AOPPs and to investigate the relationships between oxidative stress markers and clinical and biochemical parameters of cardio-metabolic risk in overweight and obese children and adolescents.

The main findings of this study are: (1) in overweight and obese children and adolescents, sRAGE was significantly lower and AOPPs and AGEs/sRAGE-ratio were significantly higher compared to controls. (2) BMI SD was an independent predictor of oxidative stress markers serum levels.

The links between AGEs, sRAGE, and AOPPs and obesity-related cardio-vascular and metabolic risk factors have been extensively investigated in adults. In particular, a significantly higher serum levels of AGEs and AOPPs and a significantly lower serum levels of sRAGE have been documented among obese adults compared to lean controls (5, 6, 19, 20). Conversely, pediatric studies about the role of oxidative stress biomarkers in obesity showed few and contrasting results. Sebekova et al., in a case-control study involving children and adolescents, reported a significantly lower plasma levels of AGEs in obese, despite higher AOPPs, in comparison with lean counterparts; however, these authors did not document any difference in sRAGE concentration (21). In a cohort of children and adolescents, Accacha et al. found an inverse relation of both sRAGE and AGEs with BMI and percent body fat (22). Other studies confirmed that obesity is associated with lower levels of sRAGE in pre-pubertal cohorts of children (23, 24) and reported that sRAGE was independently associated with liver steatosis (23) and carotid intima-media thickness (24). However, recently, other authors did not find any significant association between markers of obesity and AGEs or sRAGE (25).

Other studies reported a significantly higher AOPPs levels in obese children in comparison with controls (18, 26–28). Our findings confirmed the role of AOPPs as a trustworthy oxidative stress biomarker in obese children, showing a significant correlation of AOPPs negatively with sRAGE and positively with AGEs and AGEs/sRAGE-ratio.

In our study, we demonstrated an alteration of oxidative homeostasis expressed by lower serum levels of sRAGE and higher levels of AOPPs in overweight and obese children and adolescents, as documented in adults. Despite the difference of AGEs serum levels between groups did not reach the statistical significance, we demonstrated a significant higher values of AGEs/sRAGE-ratio in overweight/obese children compared to controls. In the assessment of oxidative homeostasis, AGEs/sRAGE-ratio has become increasingly important since it reliably expresses the relationship between oxidant factors, as AGEs and RAGE, and anti-oxidant factors, as sRAGE, glyoxilase-1, and glyoxalase-2 [two key enzymes in anti-glycation defense system (29, 30)], and AGER1, AGER2, AGER3 [receptors of AGEs involved in blocking AGEs–RAGE-mediated intracellular pathways (7)], which should be difficult-to-measure *in vivo*. An increase in AGEs/sRAGE-ratio would reveal a relative shift to oxidant from anti-oxidant factors (7). In our study, for the first time in a pediatric cohort, a significant higher value of AGEs/sRAGE-ratio among overweight and obese children and adolescents was documented.

Oxidative stress markers levels measurement, in addition to clinical and laboratory parameters evaluation, might be useful in the assessment of precocious cardio-metabolic risk of obese children; however, it is necessary to determine a specific range of risk for AGEs and sRAGE levels, through longitudinal studies, before to introduce these markers in daily clinical practice.

Moreover, in our study a significant association between BMI SD and oxidative stress markers serum levels was documented. At regression analysis BMI SD was an independent predictor of AGEs/sRAGE-ratio, sRAGE, and AOPPs serum levels, therefore the severity of overweight seems to play an important role in increasing oxidative stress in human organism, even in children. Our results support the hypothesis that overweight, independently from modifications of biochemical markers of cardio-metabolic risk, influences the alteration of the oxidative balance.

Another interesting aspect highlighted in our study was the possible influence of lipid profile alterations in oxidative dysregulation. This relation is supported by both the evidence of an independent association between TG levels and AGEs or AOPPs levels, and the demonstration of a significant association between oxidative stress markers levels and TC/HDL-ratio that is a reliable marker of cardio-metabolic risk (15, 31).

Oxidative stress promotes the inflammation through activation of intracellular cascades that ultimately determine an increased expression of pro-inflammatory cytokines and chemokines, oxygen radicals, cell adhesion molecules and acute phase proteins (7, 32, 33). Moreover, obesity itself contributes to determine a sub-clinical inflammation promoting the production of pro-inflammatory factors involved in the pathogenesis of obesity-related complications (3). According to these evidences, in our study CRP, although unspecific marker of inflammation was strongly related to oxidative stress markers levels and it was an independent predictor of AOPPs and AGEs/sRAGE-ratio.

It might be argued that our study has some limitations. First, due to the cross-sectional design of the study, we are unable to verify the causal relationships between oxidative stress markers and cardio-metabolic risk variables that could be clarified in a longitudinal study involving a further enlarged cohort of patients. Second, CRP was the only inflammatory marker considered, rather than more specific ones, as pro-inflammatory interleukins.

In conclusion, our findings suggest the presence of an AGE/RAGE-related and AOPPs-related oxidative homeostasis dysregulation that could enhance susceptibility to oxidative/inflammatory tissues damage in overweight and obese children and adolescents. Severity of overweight, influencing the increase of oxidative stress in human organism and even in children, may contribute to the pathogenesis of long-term cardiovascular and metabolic alterations.

## Ethics Statement

This study was carried out in accordance with the recommendations of Ethics Committee of Messina, with written informed consent from all subjects (parents or legal tutors). All subjects (parents or legal tutors) gave written informed consent in accordance with the Declaration of Helsinki. The protocol was approved by the Ethics Committee of Messina.

## Author Contributions

DC, MW, FD, and RR conceived the manuscript. DC, TA, and GP were involved in data collection. AA carried out data analysis. MC performed oxidative stress markers serum levels measurements. DC and MW were involved in writing of the manuscript. All authors approved the submitted version of the manuscript.

### Conflict of Interest Statement

The authors declare that the research was conducted in the absence of any commercial or financial relationships that could be construed as a potential conflict of interest.
